# Confab – generation of diverse low energy conformers

**DOI:** 10.1186/1758-2946-3-S1-P32

**Published:** 2011-04-19

**Authors:** NM O’Boyle, T Vandermeersch, GR Hutchison

**Affiliations:** 1 Analytical and Biological Chemistry Research Facility, University College Cork, Cork, Ireland; 2 University of Antwerp, Antwerp, Belgium; 3 Department of Chemistry, University of Pittsburgh, 219 Parkman Avenue, Pittsburgh, Pennsylvania 15260, USA

## 

We present a new program for conformer generation, Confab, which aims to generate diverse low energy conformers that span the space of all possible conformations. Such conformers are required for use in fields such as docking and pharmacophore searching and generation.

Confab uses a torsion driving approach to travel through the set of systematically generated conformers selecting those which are below a particular energy cutoff and which are structurally distinct (according to a user-specified RMSD) from those conformers already selected. The RMSD is evaluated using Kabsch alignment [1] of heavy atoms and takes symmetry into account. To improve performance, the alignment is carried out using the Eigen maths library [2], an open source library with an emphasis on speed and efficiency. In addition, the initial pass through the set of conformers uses a tree data structure to minimise the number of alignments required to identify a structure as similar to a previous one.

Confab is open source, and uses the Open Babel cheminformatics toolkit [3,4].
